# Supplementary figures and images for: Spatio-temporal prediction model of out-of-hospital cardiac arrest: Designation of medical priorities and estimation of human resources requirement
Source: PLoS One. 2020 Aug 31;15(8):e0238067. doi: 10.1371/journal.pone.0238067 (PMC7458314; doi:10.1371/journal.pone.0238067)

**A: All**

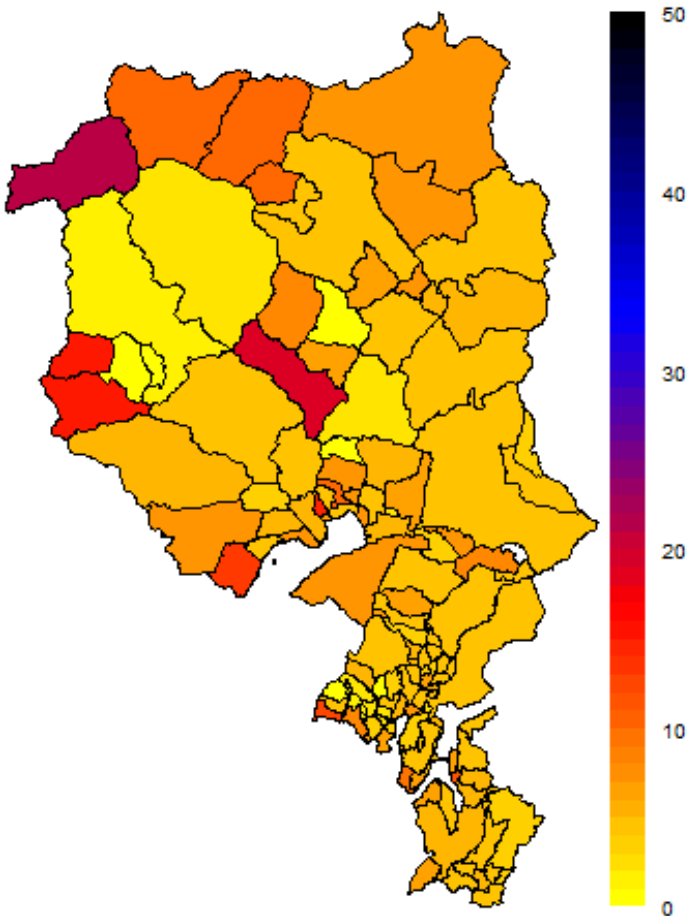

**B: Male**

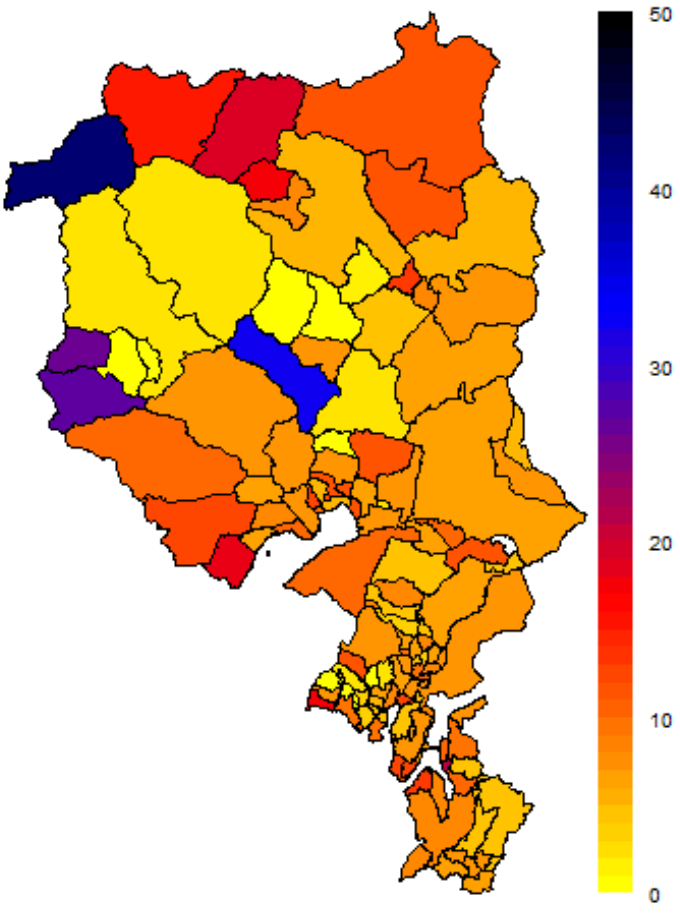

**C: Female**

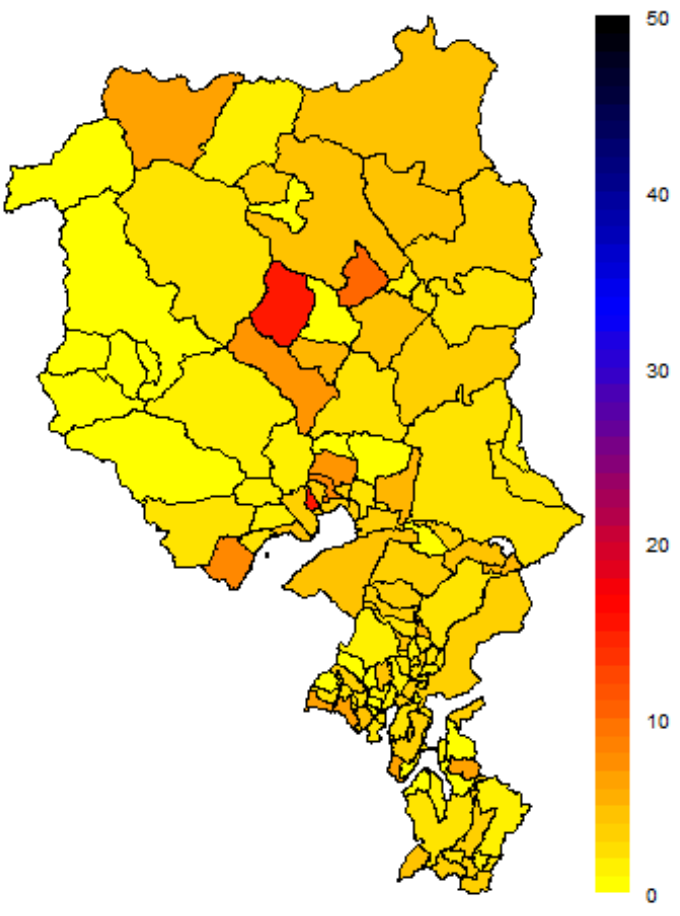

Supplement: S1 Fig — Incidence was calculated according to the inhabitants of each municipality and according to the inhabitants of each gender for each municipality for the gender-related analysis. (PDF) [file pone.0238067.s001.pdf]
