# Supplementary material for: Spatio-temporal prediction model of out-of-hospital cardiac arrest: Designation of medical priorities and estimation of human resources requirement
Source: PLoS One. 2020 Aug 31;15(8):e0238067. doi: 10.1371/journal.pone.0238067 (PMC7458314; doi:10.1371/journal.pone.0238067)
Supplement: S1 Table — (DOCX) [file pone.0238067.s002.docx]

|  | Unchanged trend | Downward trend | Upward trend | P value |
| --- | --- | --- | --- | --- |
| No. of municipalities | 109 | 4 | 4 |  |
| No. OHCA cases | 2116 | 89 | 41 |  |
| Age (yrs) | 74 [63-82] | 73 [62-79] | 78 [68-82] | 0.14 |
| Men (%) | 1490 (70.4%) | 68 (77.2%) | 28 (68.3%) | 0.36 |
| Home location OHCA (%) | 1405 (66.4%) | 60 (67.4%) | 28 (68.3%) | 0.96 |
| Time from call to EMS arrival, mins (median, IQR) | 10 [8-14] | 11 [9-14] | 13 [10-16]* | <0.001 |
| Unwitnessed OHCA (%) | 838 (39.6%) | 33 (37%) | 15 (36.6%) | 0.85 |
| Bystander CPR (%) | 1265 (59.8%) | 60 (67.4%) | 29 (70.7%) | 0.14 |
| Time from call to CPR, mins (median, IQR) | 6 [3-10] | 6 [2-10] | 6 [4-11] | 0.49 |
| First rhythm shockable (%) | 731 (35.7%) | 35 (41.7%) | 11 (26.8%) | 0.26 |
| Shock before EMS arrival (%) | 120 (5.7%) | 5 (5.6%) | 4 (9.8%) | 0.46 |
| Sustained ROSC (%) | 734 (34.7%) | 34 (38.2%) | 13 (31.7%) | 0.72 |
| Time from event to ROSC, mins (median, IQR) | 26 [19-35] | 26 [19-36] | 29 [22-37] | 0.71 |
| Survival at hospital discharge (%) | 424 (20%) | 19 (21.3%) | 12 (29.3%) | 0.31 |

*p<0.001 vs. unchanged trend in OHCA (Bonferroni correction)
